# Parental involvement, self-efficacy, and learning motivation of hearing-impaired students: insights from Savelugu, Ghana

**DOI:** 10.3389/fpsyg.2025.1705133

**Published:** 2026-02-16

**Authors:** Isaac Nyame, Mary Nyeyem Issah, Sampson Mante, Christiana Nyarko Adjei, Diana Baapeng

**Affiliations:** 1 Department of Educational Foundations Studies, Faculty of Education, University for Development Studies, Tamale, Ghana; 2 Department of Teacher Education, Kwame Nkrumah University of Science and Technology, Kumasi, Ghana

**Keywords:** deaf education, learning motivation, parental involvement, self-efficacy, special education

## Abstract

The educational development of learners with Hearing Impairment (HI) remains a significant concern within inclusive education systems, especially in low-resource countries. The influence of parental involvement and academic self-efficacy on motivation to learn is considered important, but their relationship has not been thoroughly examined in Sub-Saharan Africa. The purpose of the study was to evaluate the effect of parental involvement and self-efficacy on the learning motivation of HI students at the Savelugu School for the Deaf in Ghana. A quantitative descriptive-correlational design was used. A census approach was adopted to include all 141 students in junior high school. Data were collected using adapted scales measuring parental involvement, self-efficacy, and learning motivation. Data analysis involved descriptive statistics, Pearson correlation, and multiple regression. The authors found that parental involvement (M = 3.30, SD = 0.9), self-efficacy (M = 3.9, SD = 1.0), and learning motivation (M = 3.8, SD = 0.9) were all high. Parental involvement and self-efficacy showed a strong and significant positive correlation (*r* = 0.633, *p* < 0.001), and both variables were significant predictors of learning motivation (*R*^2^ = 0.666, *p* < 0.001), with self-efficacy (*r* = 0.523) being a slightly stronger predictor than parental involvement (*r* = 0.428). This study highlights the vital roles of parental support and self-confidence fostering in enhancing the academic motivation of students with HI. It was recommended that educational interventions should target strategies that develop learners’ self-efficacy and support active parental involvement, thereby improving learning outcomes in special education settings.

## 1 Introduction

Students with hearing impairments (HI) face particular challenges, including communication difficulties, social isolation, and limited access to assistive devices (Fadda et al., 2024; Bowen and Probst, 2023). Despite the international shift towards inclusive education, further research is needed to understand how to improve the academic performance of students with HI, particularly in resource-limited settings such as Ghana. Self-efficacy and parental involvement are often identified as important factors influencing the academic achievement of all students, especially those with disabilities (Wilder, 2023; Abodunrin, 2021). However, the specific influence of these factors on the learning motivation of HI students has not been thoroughly examined, especially within the African context.

Active parental involvement in their child’s education, encompassing support, communication, and participation in school activities, is referred to as parental involvement and has a significant impact on academic achievement (Gonzalez-DeHass and Willems, 2024; Đurišić and Bunijevac, 2017). This involvement is especially critical for learners with HI. Through parental support and engagement, communication barriers can be lowered (Hamad et al., 2022), essential resources can be accessed, and emotional support can be provided, helping them to function effectively in a world mainly inhabited by hearing individuals (Khalid et al., 2025; Đurišić and Bunijevac, 2017). Conversely, parents of children with HI also face specific challenges, such as limited proficiency in sign language and a lack of knowledge about deaf culture, which may impede effective communication and academic assistance.

Meanwhile, self-efficacy, the perceived ability of a person to learn or perform actions at a certain level, is a highly important predictor of student motivation and success (Schunk and DiBenedetto, 2022a,b; Zheng et al., 2021; Abdolrezapour et al., 2023). Students with higher self-efficacy are also more likely to volunteer for challenging tasks, work harder, and persist in the face of failure (Zhang, 2025; Meng and Zhang, 2023). A design that promotes high self-efficacy could theoretically serve as a vital protective feature for a learner with HI, who often faces multiple obstacles to academic and social success (Luo et al., 2024).

The driving forces behind academic engagement are the internal conditions that activate, guide, and sustain goal-oriented behaviours and motivation to learn (Bizimana, 2025; Wentzel, 2020). Although the connection between parental involvement, self-efficacy, and motivation has been established within hearing populations (Silinskas and Kikas, 2019; Shao and Kang, 2022), the relationship among these variables and learners with HI in Ghana remains poorly explored. Recent research, such as Agyire-Tettey et al. (2017) and Opoku et al. (2022), has highlighted that deaf education in Ghana faces systematic issues that often neglect the empirical investigation of motivational constructs within this group.

Therefore, the current study effectively addressed this gap by examining the effect of parental involvement and self-efficacy on students’ motivation to learn at the Savelugu School of the Deaf in Ghana. It is hoped that by clarifying these relationships, the findings will inform the development of specific interventions and policies to improve the educational experiences and outcomes of learners with hearing impairments in such environments.

## 2 Literature review

Parental involvement in deaf education is essential because it enables parents to understand the support available to their children, whether at home or school. It is a complex concept encompassing various behaviours, such as assisting students with homework, maintaining open communication with educators, and attending school events (Palavi et al., 2025; Gonzalez-DeHass et al., 2018). In deaf education, parental efforts to facilitate effective communication, collaborate with specialists, and connect with deaf individuals also fall within this scope (Palavi et al., 2025; Erbasi et al., 2018). Research consistently shows that parental involvement positively impacts language development (Druhan, 2023; Baker et al., 2018), academic achievement (Muitu et al., 2024; Axford et al., 2019), and the social-emotional well-being of children with hearing impairments (Yuen et al., 2022).

However, this is primarily influenced by parents’ hearing status, socioeconomic resources, and access to early intervention services (Binos et al., 2023). Parents of children with HI might face increased pressure and may require additional specialised guidance on how to effectively support their child’s education (Shastri et al., 2024; Zaidman-Zait et al., 2019). In the Ghanaian context, Opoku et al. (2022) found that interventions could be limited by communication barriers and societal negative attitudes that parents often encounter. Nonetheless, the potential for parental intervention and positive effects on academic performance remains significant (Cosso et al., 2022; Amponsah et al., 2018).

### 2.1 Student academic self-efficacy

One of the main aspects of Bandura’s (1997) social cognitive theory is self-efficacy, which posits that beliefs about one’s abilities significantly influence motivation, behaviour, and emotional outcomes (Yuen et al., 2022). Academic self-efficacy refers to a student’s confidence in their capacity to complete academic tasks and attain desired results (Chen et al., 2025; Zhang, 2025; Meng and Zhang, 2023). Students with high academic self-efficacy are more likely to set ambitious goals, work harder, adopt more effective learning behaviours, and show resilience when faced with difficulties (Huang and Kou, 2025; Zhao et al., 2024).

Among learners with HI, the development of self-efficacy may be affected by communication barriers that can obstruct mastery experiences and vicarious learning. Their self-confidence might also be harmed by negative stereotypes and lowered expectations (Hammad and Awed, 2023; Ebrahimi et al., 2015). However, research has demonstrated that HI students who receive appropriate support, such as explicit strategy training, positive feedback, and encouragement of a growth mindset, can cultivate strong self-efficacy beliefs, leading to improved academic achievement (Davenport et al., 2024; Zhang, 2025; Meng and Zhang, 2023).

Parental involvement has been reported to enhance self-efficacy. Michelle et al. (2025) found that parental involvement boosted learners’ self-efficacy by providing them with the resources, encouragement, and genuine support essential for academic success. Other researchers reported that learners with special needs are more likely to participate in learning activities, persevere through challenges, and ultimately achieve beneficial academic goals when they accept that they can overcome hindrances with the help of their parents (Masondo and Mabaso, 2025; Pham et al., 2024; Tiengsomboon and Luvira, 2024).

### 2.2 Learning motivation among hearing impaired learners

Learning motivation is a crucial factor that influences a student’s engagement, perseverance, and commitment to academic activities (Wentzel, 2020). It can be intrinsic (fostered by internal interest and enjoyment) or extrinsic (motivated by external rewards or pressures) (Morris et al., 2022). Evidence suggests that academic and social challenges, such as difficulty accessing the curriculum, repeated failures, and social isolation by peers and teachers, can hinder students’ motivation (Bowen and Probst, 2023; Alshutwi et al., 2020).

It is well established that learner motivation partly depends on parental involvement and self-efficacy. Parental support can foster a sense of belonging and emphasise the importance of education, thereby increasing motivation (Shao and Kang, 2022). Similarly, self-efficacy directly influences motivation by shaping students’ goals, effort levels, and their ability to overcome challenges (Schunk and DiBenedetto, 2022a,b). The significance of factors such as teacher support, peer acceptance, and personal self-image in affecting students’ motivation to learn with hearing impairments has been clearly emphasised (Yuen et al., 2022; Chen et al., 2020).

### 2.3 The present study

Although parental involvement, self-efficacy, and learning motivation are well-defined, their interrelationships within the specific population of HI students in Ghana remain unclear. This study aimed to address this significant gap. Based on the social cognitive theory proposed by Bandura, which suggests that the reciprocal interactions among the elements at personal, environmental, and behavioural levels are triadic (e.g., self-efficacy, parental involvement, and motivated learning), this study hypothesises that:

*H1*: Parental involvement and the self-efficacy of students have a significant positive correlation.

*H2*: Parental involvement and self-efficacy are important and positive predictors of the learning motivation of the students.

### 2.4 Contribution of the study

The study broadens Self-Determination Theory (SDT) through an empirical analysis of the interactions among environmental, personal, and behavioural factors among learners with hearing impairments in a low-resource African setting. It also enhances quantitative research in Ghana by clarifying the relationship between parental involvement, self-efficacy, and motivation among such learners. Practically, it provides evidence-based guidance to improve parental engagement and foster self-efficacy through educational interventions.

## 3 Methodology

The research used a quantitative, descriptive-correlational design. This approach was suitable because it allowed the description of parental involvement, self-efficacy, and learning motivation levels, as well as the analysis of their relationships, without any manipulation (Creswell and Creswell, 2023). A cross-sectional survey was conducted to collect data at a single point in time. The study took place at the Savelugu School for the Deaf in the Northern Region of Ghana. The school was established in 1978 as a formal institution dedicated solely to educating students with hearing impairments. The target population consisted of all students enrolled at the school during the 2024/2025 academic year, encompassing the entire Junior High School (JHS). The sample consisted of 141 students from Forms 1 to 3. This group was chosen because they possessed the necessary literacy skills to understand and complete the self-report questionnaire.

The census approach was employed to include all 141 students in the study, comprising JHS 1 (*n* = 48), JHS 2 (*n* = 61), and JHS 3 (*n* = 32). Data were collected using a questionnaire. The self-administered questionnaire consisted of four sections. The first section gathered demographic information on age, sex, and grade level. The second section included items measuring parental involvement, adapted from the Parental Involvement Rating Scale (PIRS) by Naseema and Adoo (2001). The measurements utilised a 5-point Likert Scale (1 = Strongly Disagree to 5 = Strongly Agree). In this study, the adapted scale demonstrated high internal consistency (*α* = 0.988). The third section assessed students’ self-efficacy using a 13-item scale adapted from the Morgan–Jinks Student Efficacy Scale (Jinks and Morgan, 1999). It employed a 5-point Likert scale, and the modified scale proved to be highly reliable (*α* = 0.977). The motivation items were based on the existing theories and measures in the educational psychology, such as Self-Determination Theory (Ryan and Deci, 2020), the Motivated Strategies to Learning Questionnaire (Pintrich et al., 1991), Self-Regulated Learning theory (Zimmerman, 2002), and the Social Cognitive Theory (Bandura, 1997). It used a 5-point Likert scale, which showed good scale reliability (*r* = 0.975). The instruments were translated to ensure clarity and cultural relevance. Face and content validity were assessed through reviews by specialists in special education and a pilot study involving 15 students.

The school administration was approached and approved the study. Participants were informed of the study’s purpose beforehand, and consent was obtained by completing the questionnaire. Communication needs were also addressed by having a qualified sign language interpreter present during the group administration to clarify instructions and questions. The questionnaires were completed in a classroom setting, and respondents were assured of the confidentiality and anonymity of their responses.

Statistical Package for the Social Sciences (SPSS) version 25.0 was utilised to analyse the data. The demographic data were examined using descriptive statistics (frequencies, percentages, means, and standard deviations) to summarise the Likert-scale responses on the variables. Hypothesis 1 (the correlation between parental involvement and self-efficacy) was tested using Pearson product–moment correlation. Multiple linear regression analysis was performed to evaluate Hypothesis 2 (the predictive effects of parental involvement and self-efficacy on learning motivation), with the significance level set at *p* < 0.05.

## 4 Results

The study achieved a 100% response rate (*N* = 141). Table 1 shows that the sample included more males (61%, *n* = 86) than females (39%, *n* = 55). The majority of participants (67%, *n* = 95) were aged 15–18 years. There was proportional representation of students in JHS Form 2 (43%, *n* = 61), Form 1 (34%, *n* = 48), and Form 3 (23%, *n* = 32).

**Table 1:** Correlation analysis results.

Variables	Statistics	Parental involvement	Self-efficacy
Parental involvement	Pearson correlation	1	0.633
	Sig. (2-tailed)		0.000
	*N*	141	141
Self-efficacy	Pearson correlation	0.633	1
	Sig. (2-tailed)	0.000	
	*N*	141	141

**Denotes level of significance of the correlation coefficient. Correlation significant at the 0.01 level (2-tailed).

The descriptive analysis indicated that students scored highly across the three constructs. Parental involvement had an average of 3.30 (SD = 0.9) on the 5-point scale. Some areas with strong involvement included parents providing extra attention during exams (M = 4.6, SD = 0.6), frequently asking about school lessons (M = 4.5, SD = 0.6) and engaging with study schedules (M = 4.2, SD = 0.9). However, participation in Parent-Teacher Association (PTA) events was very low (M = 2.6, SD = 1.5). Since the school is residential with no day students, it is unclear how the students perceived their parents’ direct involvement in their academics, as found in this study, and this requires further exploration.

Students’ self-efficacy was high (M = 3.9, SD = 1.0). They were quite confident in their ability to learn class content (M = 4.3, SD = 0.9), in their future career success (M = 3.9, SD = 1.0), and in their belief that effort pays off (M = 3.6, SD = 1.1). The motivation for learning was also high (M = 3.8, SD = 0.9). Students expressed a strong belief in their growth potential (M = 4.5, SD = 0.7), recognised the need to keep learning (M = 3.8, SD = 1.0), and believed in their persistence to work hard (M = 3.8, SD = 1.0).

### 4.1 Analysis of hypotheses

Table 1 illustrates the relationships among the variables as proposed in the first three hypotheses. Hypothesis 1 predicted a significant positive correlation between parental involvement and self-efficacy. The Pearson correlation analysis confirmed this hypothesis. The two variables showed a positive and significant correlation (*r* = 0.633, *n* = 141, *p* < 0.001). This suggests that the greater the perceived parental involvement, the higher the academic self-efficacy among HI students, as shown in Table 1.

Hypothesis 2 proposed that parental involvement and self-efficacy would act as significant predictors of learning motivation. Learning motivation was the dependent variable in a multiple regression analysis. As summarised in Table 2, the results indicate that the model was significant, *F*(2, 138) = 137.307, *p* < 0.001, and accounted for 66.6% of the variance in learning motivation (R^2^ = 0.666, Adjusted R^2^ = 0.661).

**Table 2:** Regression analysis results of parental involvement, students’ self-efficacy and learning motivation.

Model summary				
Model	*R*	*R* square	Adjusted *R* square	Std. error of the estimate
1	0.816	0.666	0.661	0.65404

ANOVA^a^						
Model		Sum of squares	*df*	Mean square	*F*	Sig.
1	Regression	117.471	2	58.736	137.307	0.000
	Residual	59.032	138	0.428		
	Total	176.504	140			

Model		Unstandardized coefficients		*t*	Sig.
		*B*	Std. error		
1	(Constant)	0.793	0.210	1.277	0.204
	Parental involvement	0.428	0.071	6.039	0.000
	Self-efficacy	0.523	0.064	8.122	0.000

Learning motivation was significantly predicted by parental involvement (*b* = 0.428, *t* = 6.039, *p* < 0.001) and self-efficacy (*b* = 0.523, *t* = 8.122, *p* < 0.001). The standardised beta coefficients indicate that self-efficacy had a slightly greater influence on predicting learning motivation compared to parental involvement.

^a^Represents predictors in the regression model which were self-efficacy and learning motivation.

## 5 Discussion

This study aimed to explore the relationships between parental involvement, self-efficacy, and motivation to learn among HI students in Ghana. The findings provide compelling evidence of how these factors are vital to the academic lives of this group. Parental involvement levels were high (M = 3.30), which is very encouraging given the limited resources within families of persons with disabilities. This result contrasts with some sources that suggest parents of children with HI in low-income countries may be less engaged due to communication difficulties and a lack of support (Opoku et al., 2022). The high scores on questions about academic support at home (e.g., giving extra attention, asking about lessons) indicate that parents are actively involved in their children’s education. However, the lower score in PTA participation aligns with previous studies and suggests a possible disconnect between home-based and school-based involvement. This might be due to logistical issues, parents’ comfort levels in the school environment, communication barriers, or the absence of school-led initiatives to facilitate such events (e.g., providing sign language interpreters).

Hypothesis 1 is supported by the strong and positive relationship between parental involvement and self-efficacy (*r* = 0.633, *p* = 0.001), consistent with social cognitive theory (Yuen et al., 2022; Bandura, 1997). Involved parents provide encouragement, demonstrate problem-solving behaviours, and create environments rich in mastery experiences, which are key sources that can foster self-efficacy (Zhang, 2025; Meng and Zhang, 2023; Shebani et al., 2025; Davenport et al., 2024). For an HI student, a parent’s willingness to communicate and support their learning despite challenges reflects a belief in the child’s abilities, which, in turn, boosts the child’s self-confidence (Yuen et al., 2022). This suggests that parental support goes beyond academic assistance, playing a crucial role in fostering the child’s psychological resilience.

The regression analysis strongly supported Hypothesis 2, and the combined confidence in the predictive ability of parental involvement and self-efficacy explained 66.6% of the variance in learning motivation, which is a substantial percentage. This figure indicates that these two factors are among the most important determinants of whether a HI student feels motivated to learn. Notably, the prediction of self-efficacy (*b* = 0.523) was slightly more accurate than that of parental involvement (*b* = 0.428). This suggests that, while external parental support is crucial (Shao and Kang, 2022), internalising that support into personal beliefs of competence and confidence is even more directly influential in prompting behaviour (Schunk and DiBenedetto, 2022a,b). Essentially, parental involvement acts as the fuel, and the engine transforms it into motivation to learn.

The significance of this finding for intervention is substantial. It extends beyond merely encouraging parents to become more involved and emphasises that the quality of engagement must be intentionally designed to build self-efficacy. Parents and teachers should focus on providing specific, process-oriented praise, creating situations that enable the child to achieve small victories, and helping the child recognise that their successes largely result from their effort and strategies, rather than solely from external assistance from parents (Michelle et al., 2025).

## 6 Conclusion and implications

The paper presents strong empirical evidence from the Ghanaian context that parental involvement and academic self-efficacy are closely linked, and they mutually influence learning motivation in HI students. The findings not only address complex issues but also highlight realistic psychological and social factors that should be taken into consideration. The implications of this are twofold. In practice, schools with deaf and inclusive education units must develop systematically organised programmes to empower parents. This includes:

1. *Parent education workshops*: Teaching parents to use sign language, positive academic support methods, and how to foster self-efficacy (e.g., growth mindset parenting).
2. *Enhancing school-home communication*: This can be achieved by developing simple communication methods (e.g., visual newsletters, translated PTA meetings) to bridge the gap between home-based and school-based engagement.
3. *Incorporating self-efficacy building in pedagogy*: Educators must be taught to design lessons that include mastery experiences, utilise peer models, and provide feedback focused on effort and strategy.

In terms of policy, the findings emphasise the importance of including parental support and psycho-educational elements in national models of special education. The costs for parent liaison officers, sign language interpreters at school events, and even training teachers in motivational theories are not additional expenses but vital investments in the educational outcomes of HI students.

Ultimately, a supportive and collaborative ecosystem where proactive parental involvement consistently boosts student self-efficacy can empower educators and families to nurture and sustain strong, lasting motivation to learn in deaf and hard-of-hearing students. This helps them overcome barriers and realise their full academic potential. These factors are important, co-predictors of learning motivation among HI students. The findings shift the focus further; instead of merely highlighting problems, they identify the psychological and social levers that can be used to improve the situation.

### 6.1 Limitations and future research directions

This research has limitations despite its contributions. First, relying on self-reports from students may cause common bias. Future research should utilise multiple sources, including those of observers, parents, and teachers. Second, the cross-sectional design reveals relationships but does not establish causality. Longitudinal studies are needed to see how variables influence each other over time. Third, the study’s setting in one special school limits the generalisability to HI students in inclusive schools or different cultures. Future research should include more diverse samples. Lastly, qualitative studies could better explore behaviours and interactions that support and boost confidence in HI students.

## Data Availability

The raw data supporting the conclusions of this article will be made available by the authors, without undue reservation.
